# Anion Binding by Macrocyclic Receptors: Computational Landscape of 1:1 and 2:1 Stoichiometries

**DOI:** 10.1002/jcc.70270

**Published:** 2025-11-14

**Authors:** Minwei Che, Amar H. Flood, Krishnan Raghavachari

**Affiliations:** ^1^ Department of Chemistry Indiana University Bloomington Indiana USA

## Abstract

Macrocyclic receptors play a crucial role in supramolecular chemistry, enabling the selective binding of guest molecules through non‐covalent interactions. This study investigates the anion binding properties of three shape‐persistent macrocycles, triazolophane, cyanostar, and tricarb, each featuring preorganized cavities with polarized CH hydrogen bond donors. In the 1:1 binding stoichiometries, the macrocycles preferentially bind anions that fit inside their two‐dimensional cavities, with this preference being more pronounced in solution. Specifically, the triazolophane favors smaller anions like chloride and bromide, while the larger cavities offered by cyanostar and tricarb macrocycles preferentially bind the larger iodide. For large, polyatomic anions (SCN^−^, BF_4_^−^, ClO_4_^−^, and PF_6_^−^), the macrocycles self‐assemble to form stable 2:1 sandwich complexes, exhibiting stronger binding. This shift in selectivity highlights the versatility of the macrocycles, making them promising candidates for anion sensing and regulation with various complexation stoichiometries.

## Graphical abstract

Macrocyclic receptors with versatile binding stoichiometries. 1:1 complexes for small anions and 2:1 “sandwich” assemblies for large, weakly coordinating anions.

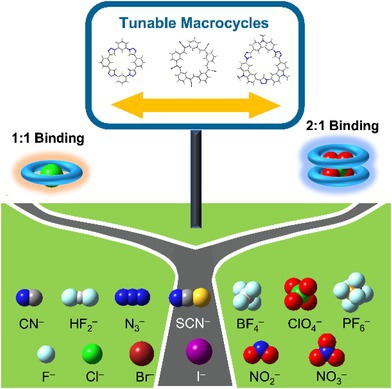

## 1 Introduction

**FIGURE 1:**
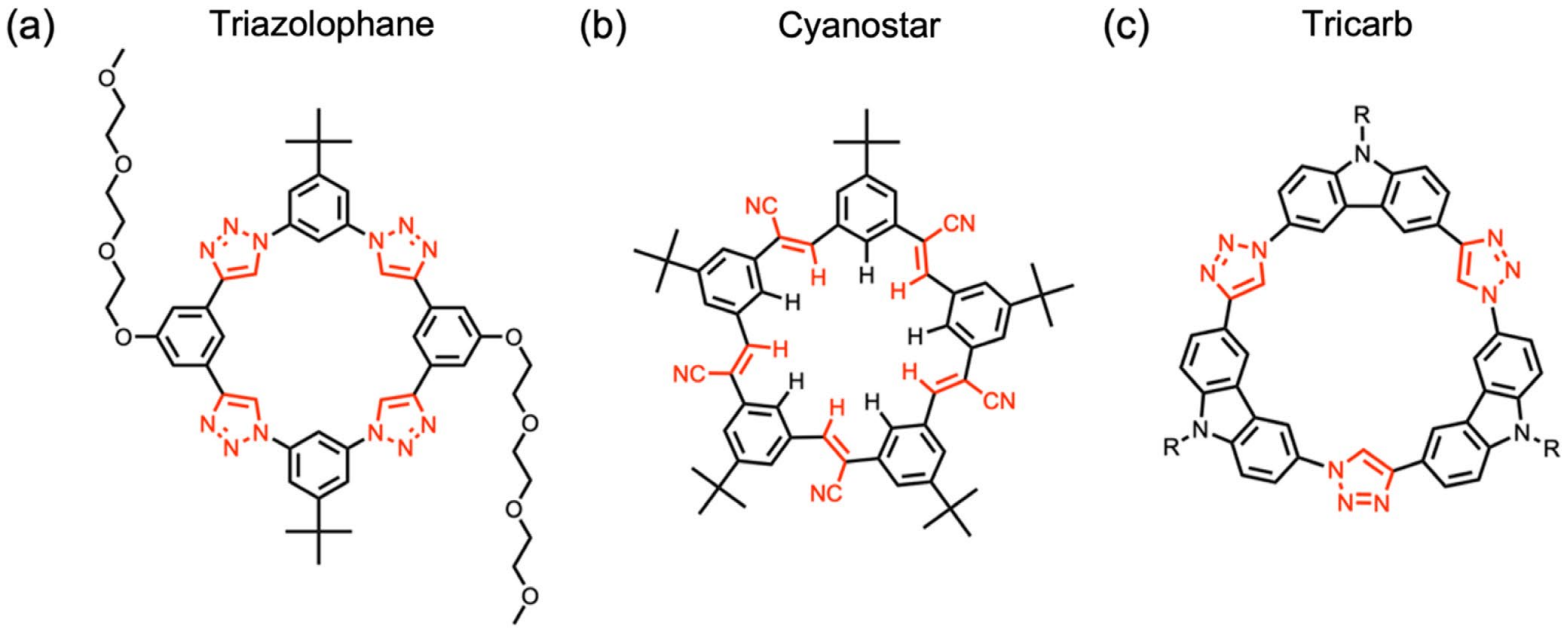
Structure of triazolophane (**Tz**), cyanostar (**CS**), and tricarb (**Tc**) macrocycles.

The development of macrocycle receptors has been central to supramolecular chemistry since the development of Pederson's crown ethers [1, 2, 3], Lehn's cryptands [4, 5, 6, 7], and Cram's spherands [8, 9, 10]. These host molecules, characterized by their structured intramolecular cavities and guest‐binding functional groups, achieve high selectivity and strong binding via non‐covalent interactions [11, 12]. Among them, anion receptors have been extensively studied due to the ubiquitous presence of anions in nature [13, 14, 15, 16]. Traditionally, anion receptors have relied on NH [14, 17, 18, 19] and OH [20, 21, 22] based hydrogen bond donors. Studies on CH hydrogen bonding were pioneered by Sutor [23] in the 1960s, though they have been traditionally regarded as weak [24]. More recently, Flood and coworkers have developed preorganized anion receptors containing polarized CH donors [25, 26, 27, 28, 29, 30, 31]. Triazolophane (**Tz**, Figure 1a) [25], cyanostar (**CS**, Figure 1b) [29], and tricarb (**Tc**, Figure 1c) [30] are three different macrocycles exemplifying this design. They feature preorganized cavities with alternating units of both strongly and weakly polarized CH donors. These structural elements contribute to both anion binding and shape persistence, making these macrocycles ideal platforms for investigating the factors governing anion selectivity.

Computational methods have been developed to enhance the understanding of non‐covalent interactions. To calculate the interaction energy, a conceptually straightforward approach is to determine the energy difference between the complex and the sum of the energies of the individual molecules, often host and guest [32]. The accuracy of this approach depends on the computational methods employed. For example, while coupled‐cluster theory [33] provides highly accurate results, its steep computational scaling makes it prohibitively expensive for large supramolecular systems. Density functional theory (DFT) [34] has become the workhorse in computational chemistry for electronic structure calculations by offering a balance between accuracy and cost. Alternatively, perturbative methods treat the interaction as a perturbation to monomer Hamiltonians [35]. Among these, SAPT [36, 37, 38] is one of the most prominent perturbative methods. It offers the advantage of directly calculating the intermolecular interaction energy, providing physically interpretable components, including electrostatics, induction, dispersion, and exchange repulsion. These energy terms align with chemical intuition, providing valuable insights into the nature of non‐covalent interactions.

Computational studies on anion receptors have largely been conducted in the gas phase. For example, Hay et al. [14] demonstrated that for urea‐based macrocycles, binding strength can be tuned by adjusting cavity size, spatial arrangement, and the number of urea groups. Valiev et al. [39] studied aromatic and antiaromatic porphyrinoids, showing that anion complexation can alter macrocyclic aromaticity. Ramabhadran et al. investigated the strong binding of cyanide [40, 41] to triazolophane and used computer‐aided design to enhance the macrocycle's affinity to bifluoride [42]. Sengupta et al. [43] analyzed the contributions of attractive forces, that is, electrostatics, induction, and dispersion, to the binding energy of macrocyclic receptors with various anions. While these studies offer valuable mechanistic insights, real‐world anion recognition occurs in solution, where solvation effects play a crucial role. To account for the solvent effects, Liu et al. [44] conducted a combined experimental and computational study on chloride binding to triazolophane across a wide range of solvents (*ɛ*_r_ = 4.7–56.2), revealing an important inverse dielectric dependence of binding energy. Debnath et al. [45] illustrated the impact of solvent polarity on the folding behavior of an aryl‐triazole foldamer.

This study explores the anion binding properties of three macrocyclic receptors: triazolophane (**Tz**), cyanostar (**CS**), and tricarb (**Tc**). Both DFT and SAPT are employed to quantify binding affinities and dissect the nature of the underlying non‐covalent interactions. The 1:1 binding properties of the three receptors are examined with 13 anions of diverse shapes and sizes: spherical (F^−^, Cl^−^, Br^−^, I^−^), linear (CN^−^, HF_2_^−^, N_3_^−^, SCN^−^), bent (NO_2_^−^), trigonal planar (NO_3_^−^), tetrahedral (BF_4_^−^, ClO_4_^−^), and octahedral (PF_6_^−^). We systematically probe the interplay between the macrocycle and the anion to determine their combined effect on binding strength. Additionally, the 2:1 binding with four large anions (SCN^−^, BF_4_^−^, ClO_4_^−^, PF_6_^−^) as well as three smaller atomic anions (Cl^−^, Br^−^, I^−^) is analyzed. The selectivity of the macrocycles in both 1:1 and 2:1 binding modes is assessed in dichloromethane (*ε*_r_ = 8.93).

## 2 Computational Methods

DFT calculations were performed using the *Gaussian 16* suite of programs [46] with the M06‐2X functional [47]. The conductor‐like polarizable continuum model (CPCM) [48] was used to account for solvent effects. For 1:1 binding, geometry optimizations were performed using the 6‐311+G(d) basis set [49, 50]. A customized in‐house basis set of comparable quality was used for iodine, with the coefficients provided in the Supporting Information. For 2:1 binding, a mixed basis set approach was employed to reduce the computational cost: 6‐311G for the atoms of the macrocycle receptors, and 6–311 + G(d) for the atoms of the anion guests. All optimized structures were verified as minima without imaginary frequencies. Single‐point energies were obtained with larger basis sets to compute the binding affinities. In these calculations, the 6–311++G(3df,2p) basis set was used for the cage atoms, except for bromine and iodine atoms, for which the *aug*‐cc‐pVTZ‐PP basis set [51, 52] containing pseudopotentials describing the inner core orbitals was used.

SAPT analysis was performed using the scaled SAPT0 method, sSAPT0 [53] implemented in the *Psi4* software [54]. It incorporates scaling factors to improve the accuracy of exchange‐type terms. The jun‐cc‐pVDZ basis set [55] was used for all atoms except for bromine and iodine for which the jun‐cc‐pVDZ‐PP basis set with pseudopotentials was applied.

## 3 Results and Discussion

**FIGURE 2:**
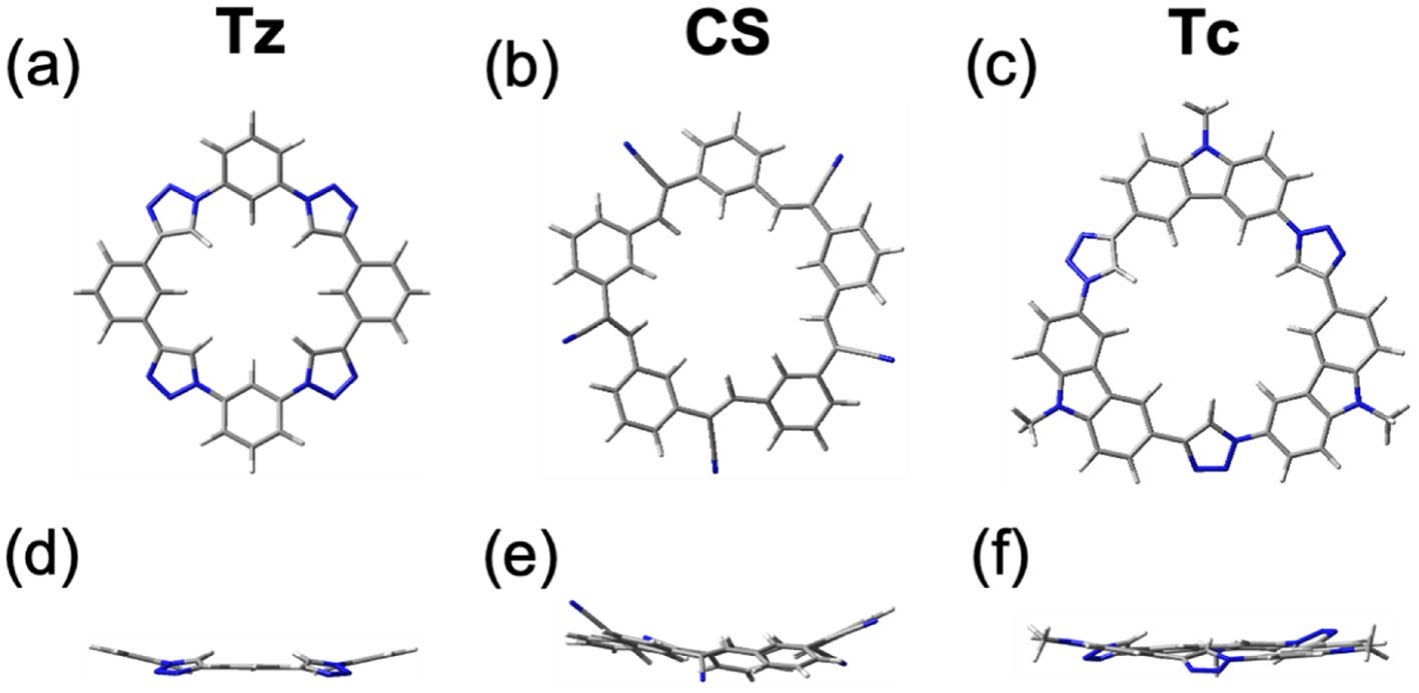
Optimized geometries for the macrocycles. Top (a–c) and side (d–f) views of (a, d) triazolophane (**Tz**), (b, e) cyanostar (**CS**), and (c, f) tricarb (**Tc**).

Triazolophane (Figure 2a) consists of alternating phenylene and highly polarized five‐membered 1,2,3‐triazole [56] units. It adopts a symmetrical square‐shaped structure with a pseudo‐circular 3.6‐Å cavity. Its CH donors are categorized into three types: north–south N‐phenylene CH donors, east–west C‐phenylene CH, and triazole CH donors at diagonal positions (H_NE_, H_NW_, H_SE_, and H_SW_). To minimize electrostatic repulsions among electropositive H atoms, triazolophane adopts a slightly puckered conformation (Figure 2d). Cyanostar is a shallow bowl‐shaped (Figure 2e) pentagonal macrocycle. With alternating phenylene and olefin CH donors (Figure 2b), it offers a 4.4 Å cavity. It shows a higher level of flexibility compared to triazolophane and adopts a shallow bowl‐shaped structure (Figure 2e). In a detailed conformational study, Liu et al. [57] found that the tilting olefins introduce flexibility while phenylenes contribute to shape persistence. Finally, tricarb (Figure 2c) features alternating carbazole and triazole units forming a 4.6 Å cavity, with nine CH donors (three from triazoles and six from carbazoles). Like triazolophane, tricarb exhibits slight puckering (Figure 2f) to reduce intramolecular repulsions.

### 3.1 1:1 Anion Binding Complexes

To assess the anion‐binding selectivity of the three macrocycles, 1:1 binding with a diverse set of 13 anions of varying shapes and sizes is investigated: F^−^, Cl^−^, Br^−^, I^−^, CN^−^, HF_2_^−^, N_3_^−^, SCN^−^, NO_2_^−^, NO_3_^−^, BF_4_^−^, ClO_4_^−^, and PF_6_^−^. These anions (Table S1) are categorized by dimensionality: 0D (spherical), 1D (linear), 2D (planar), and 3D (tetrahedral and octahedral). The selected anions span a wide size (diameter) range, from the smallest F^−^ (2.5 Å) to the largest PF_6_^−^ (4.8 Å) [58].

**FIGURE 3:**
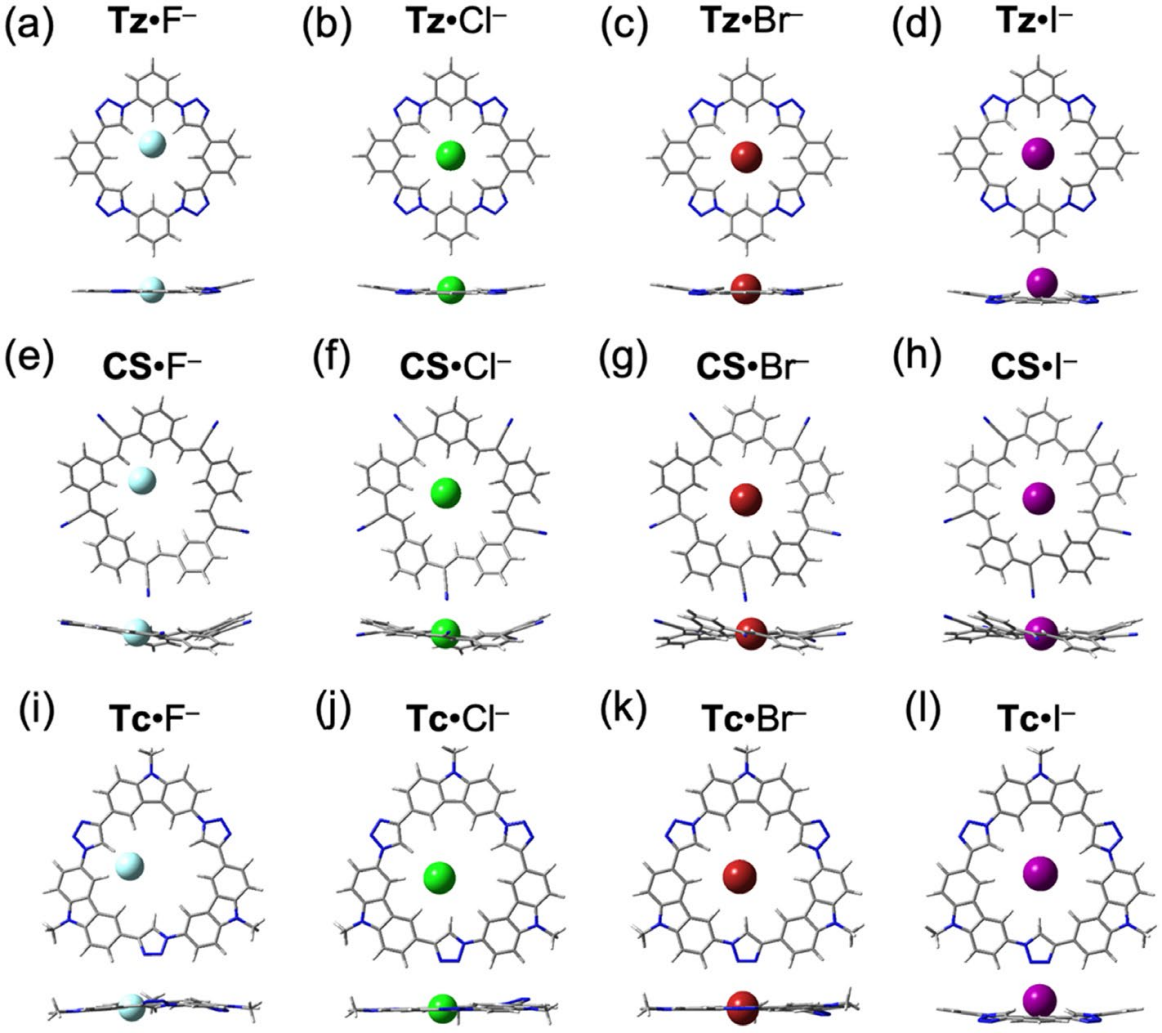
Optimized geometries for the 1:1 halide complexes with macrocycles. Top views (top row) and side (bottom row) views of (a) **Tz**·F^−^, (b) **Tz**·Cl^−^, (c) **Tz**·Br^−^, (d) **Tz**·I^−^, (e) **CS**·F^−^, (f) **CS**·Cl^−^, (g) **CS**·Br^−^, (h) **CS**·I^−^, (i) **Tc**·F^−^, (j) **Tc**·Cl^−^, (k) **Tc**·Br^−^, and (l) **Tc**·I^−^.

Size selectivity plays a key role in the 1:1 binding of spherical halides. Anions that closely match the macrocycles' cavity sizes reside snugly at the center of the binding sites, maximizing hydrogen bond interactions with the receptors (Figure 3b,c,h,l). In contrast, halides significantly smaller than the cavities adopt off‐center positions, stabilized by fewer hydrogen bonds (Figure 3a,e–g,i–k). Notably, complexation induces increased planarity in certain regions of the macrocycles where the anions reside. For instance, in **Tz**·F^−^ (Figure 3a), fluoride is positioned closest to the north N‐phenylene, and lies coplanar with the north, east and west phenylenes, as well as the northeast and the northwest triazoles. However, the southern hydrogen bond donors remain slightly puckered to reduce repulsions. In **Tz**·I^−^ (Figure 3d), where the guest anion is too big for the triazolophane cavity, iodide sits above the plane, interacting primarily with the triazoles.

**FIGURE 4:**
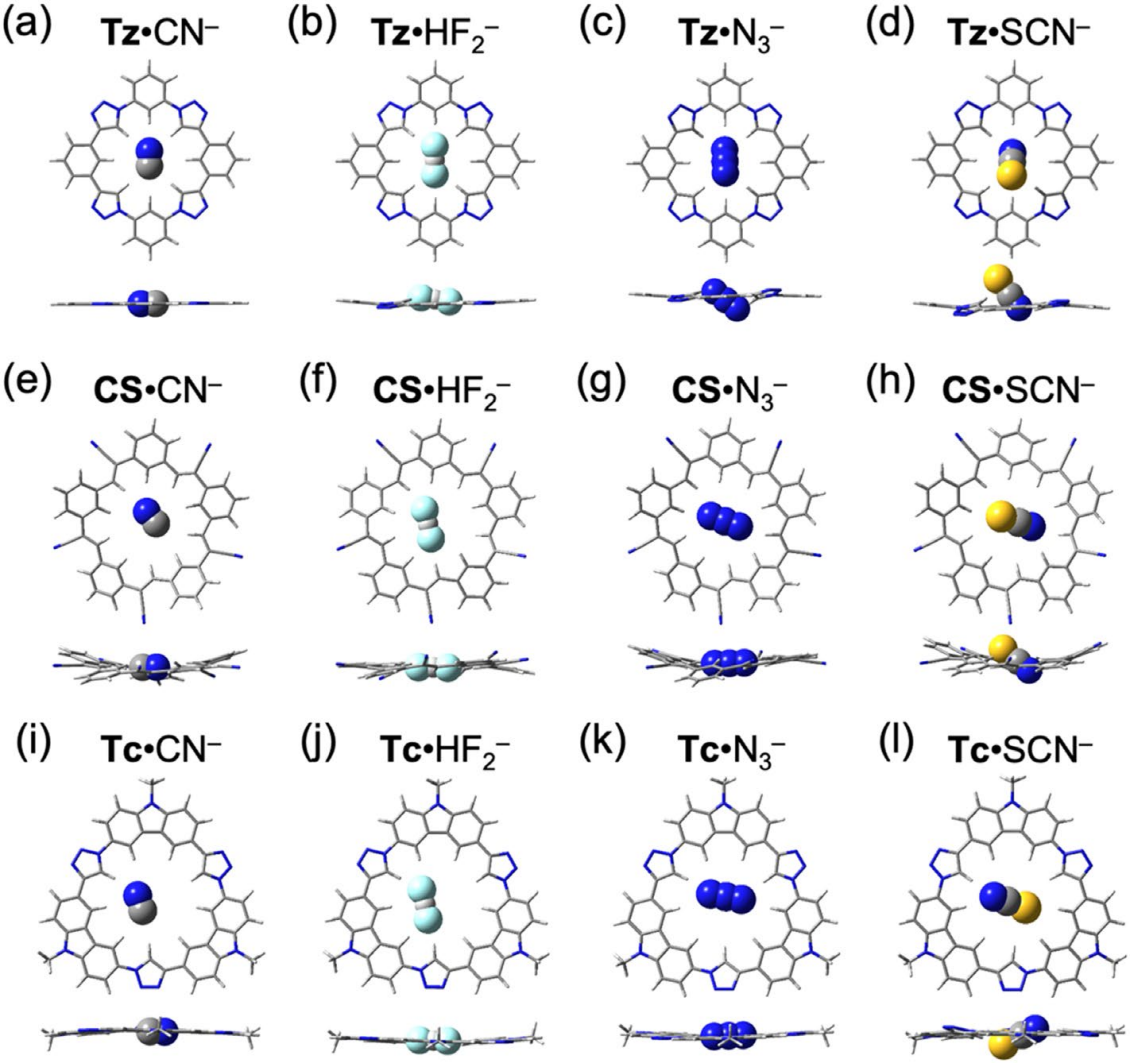
Optimized geometries for the 1:1 halide complexes with macrocycles. Top views (top row) and side views (bottom row) of (a) **Tz**·CN^−^, (b) **Tz**·HF_2_^−^, (c) **Tz**·N_3_^−^, (d) **Tz**·SCN^−^, (e) **CS**·CN^−^, (f) **CS**·HF_2_^−^, (g) **CS**·N_3_^−^, (h) **CS**·SCN^−^, (i) **Tc**·CN^−^, (j) **Tc**·HF_2_^−^, (k) **Tc**·N_3_^−^, and (l) **Tc**·SCN^−^.

The shape of linear anions enables directional hydrogen bonding with the macrocycles. In 1:1 complexes formed with triazolophane, the north–south orientation of anions is more energetically favored. Cyanide, which closely matches the small cavity of triazolophane, remains coplanar with the receptor (Figure 4a). However, larger linear anions exhibit increasing degrees of tilting out of the mean plane of **Tz**, with bifluoride tilting at 20° (Figure 4b), azide at 40° (Figure 4c), and thiocyanate at 60° (Figure 3d). This tilting is expected to weaken binding in solution, where solvation stabilizes the anions' excessive charge, making them less available for hydrogen bonding [42]. In 1:1 complexes formed with cyanostar and tricarb, CN^−^, HF_2_^−^, and N_3_^−^ are accommodated within the cavities (Figure 4e–g,i–k). Nevertheless, SCN^−^ shows noticeable tilting of 40° in **CS**·SCN^−^ (Figure 4h) and 30° in **Tc**·SCN^−^ (Figure 4l) due to the size mismatch. The observed tilting further highlights the shape persistence of the three macrocycles, as the structures of **Tz**, **CS**, and **Tc** dictate how anions adapt within their cavities.

**FIGURE 5:**
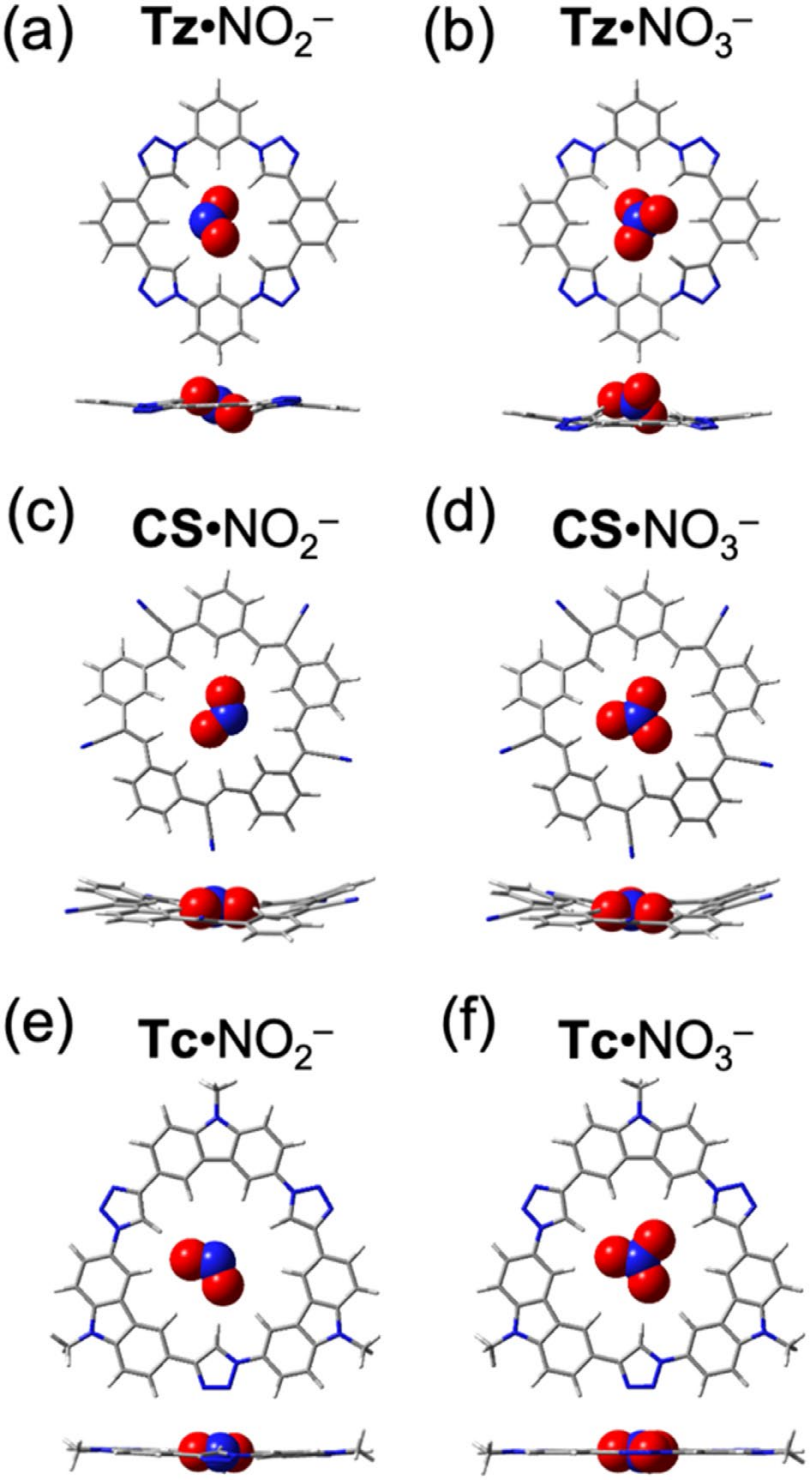
Optimized geometries for the 1:1 halide complexes with macrocycles. Top views (top row) and side (bottom row) views of (a) **Tz**·NO_2_^−^, (b) **Tz**·NO_3_^−^, (c) **CS**·NO_2_^−^, (d) **CS**·NO_3_^−^, (e) **Tc**·NO_2_^−^, and (f) **Tc**·NO_3_^−^.

For the planar NO_2_^−^ and NO_3_^−^ anions, the triazolophane cavity is too small to fully accommodate them. Consequently, both anions exhibit a 30° tilt relative to the mean plane. However, a key difference is observed: in **Tz**·NO_2_^−^ (Figure 5a), the central nitrogen remains in‐plane, while the oxygen atoms, aligned with the north and south phenylenes, are positioned off‐plane. In contrast, in **Tz**·NO_3_^−^ (Figure 5b), the anion does not enter the cavity but instead rests above the macrocycle. In comparison, both cyanostar and tricarb provide a better geometric fit, allowing NO_2_^−^ and NO_3_^−^ to be fully accommodated within their cavities without significant tilting (Figure 5c–f).

**FIGURE 6:**
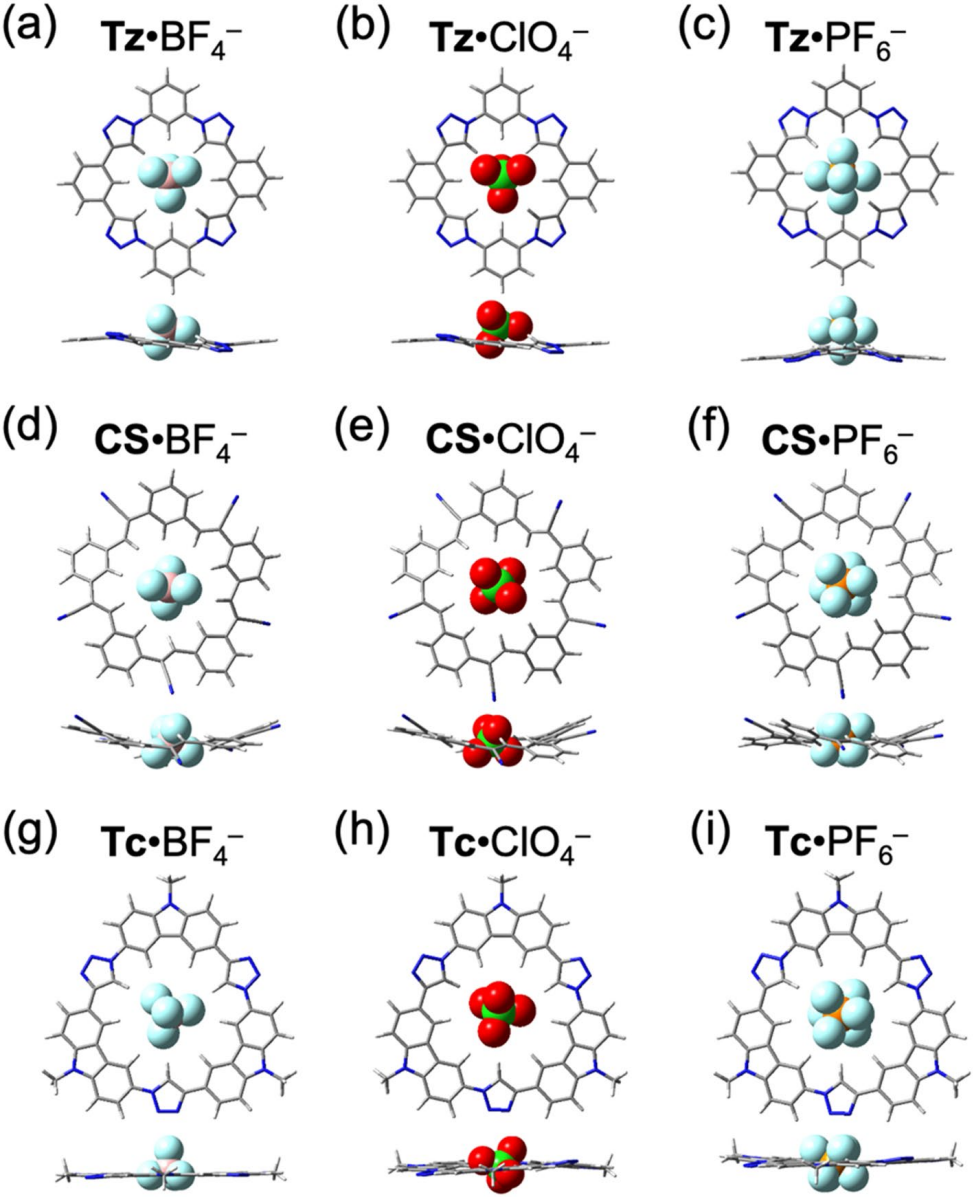
Optimized geometries for the 1:1 halide complexes with macrocycles. Top views (top row) and side (bottom row) views of (a) **Tz**·BF_4_^−^, (b) **Tz**·ClO_4_^−^, (c) **Tz**·PF_6_^−^, (d) **CS**·BF_4_^−^, (e) **CS**·ClO_4_^−^, (f) **CS**·PF_6_^−^, (g) **Tc**·BF_4_^−^, (h) **Tc**·ClO_4_^−^, and (i) **Tc**·PF_6_^−^.

Three‐dimensional anions pose a challenge for 1:1 binding with the macrocycles due to the geometry mismatch. In the smallest triazolophane, BF_4_^−^, ClO_4_^−^, and PF_6_^−^ remain largely outside the binding pocket (Figure 6a–c). While cyanostar and tricarb can accommodate these three 3D anions, their encapsulation of the anions is incomplete with the terminal atoms extending out of the macrocycle plane (Figure 6d–f,h,i). An exception is observed in **Tc**·BF_4_^−^, where the size of the tetrahedral BF_4_^−^ (4.0 Å) allows three fluorine atoms to fit within tricarb's 4.6 Å cavity (Figure 6g).

The structural analysis of the 1:1 complexes highlights the critical role of size and shape complementarity in anion binding interactions. Smaller anions adopt off‐center positions: those that closely match the macrocycle cavities form optimal hydrogen bonds, while larger anions either tilt or perch from outside the cavities. Similarly, three‐dimensional anions face challenges in encapsulation. These findings underscore the relationship between complementarity and anion selectivity that governs the 1:1 binding behavior.

**FIGURE 7:**
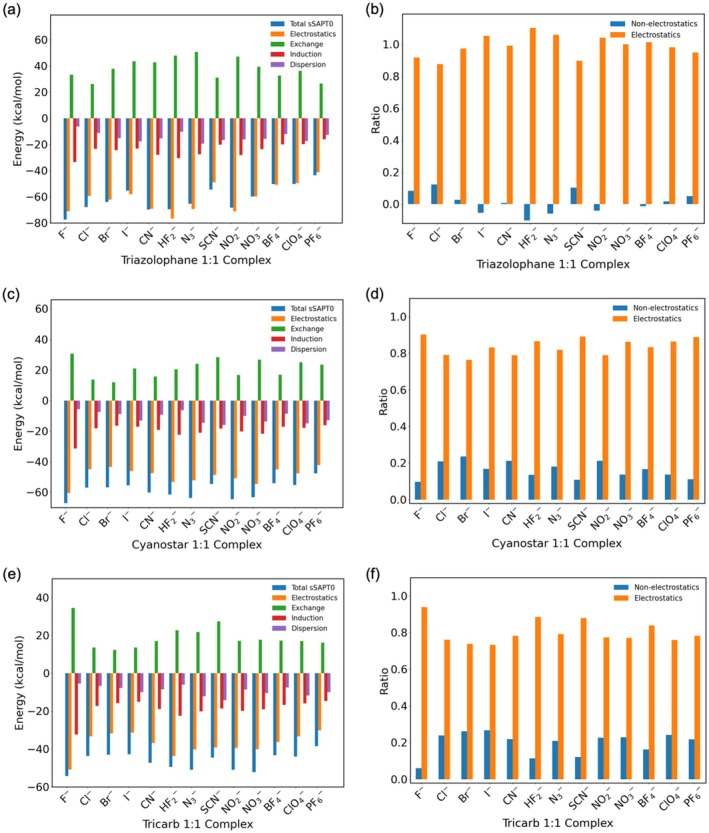
SAPT energy decomposition of 1:1 complexes for (a) triazolophane, (c) cyanostar, and (e) tricarb. Ratios of non‐electrostatics (exchange‐repulsion, induction and dispersion) and electrostatics of the total sSAPT0 energies for (b) triazolophane, (d) cyanostar, and (f) tricarb.

To evaluate the binding affinities of triazolophane, cyanostar, and tricarb, we analyzed the binding free energies (Δ*G*) in both the gas phase and DCM, as summarized in Table 1. These thermodynamic parameters were complemented by SAPT energy decomposition (Figure 7) to provide insights into the physical components of the interaction.

**TABLE 1:** Calculated 1:1 binding free energies (∆*G*, kcal/mol) for triazolophane, cyanostar, and tricarb in the gas phase and dichloromethane (DCM), along with solvation free energies of the complexes.

	${∆G}_{1:1}^{\mathrm{gas}}$	${∆G}_{1:1}^{\mathrm{DCM}}$	$∆G^{\text{solvation}}$
**Tz**·F^−^	−66.4	−14.0	−44.1
**Tz**·Cl^−^	−54.2	−11.8	−40.1
**Tz**·Br^−^	−50.7	−10.1	−37.4
**Tz**·I^−^	−43.1	−7.3	−37.5
**Tz**·CN^−^	−53.9	−12.0	−37.5
**Tz**·HF_2_^−^	−54.5	−9.8	−37.3
**Tz**·N_3_^−^	−46.1	−9.2	−38.6
**Tz**·SCN^−^	−39.5	−7.4	−38.7
**Tz**·NO_2_^−^	−50.0	−8.9	−37.1
**Tz**·NO_3_^−^	−44.3	−7.1	−37.9
**Tz**·BF_4_^−^	−39.3	−5.8	−38.6
**Tz**·ClO_4_^−^	−37.2	−5.6	−38.7
**Tz**·PF_6_^−^	−36.3	−6.3	−37.8
**CS**·F^−^	−55.8	−6.3	−45.3
**CS**·Cl^−^	−45.2	−5.9	−41.3
**CS**·Br^−^	−44.3	−5.9	−37.8
**CS**·I^−^	−42.2	−6.8	−36.1
**CS**·CN^−^	−42.9	−4.1	−38.7
**CS**·HF_2_^−^	−46.9	−4.9	−38.2
**CS**·N_3_^−^	−44.5	−5.8	−34.9
**CS**·SCN^−^	−37.1	−4.7	−36.6
**CS**·NO_2_^−^	−45.8	−4.8	−35.3
**CS**·NO_3_^−^	−45.2	−5.7	−33.6
**CS**·BF_4_^−^	−40.7	−5.6	−35.3
**CS**·ClO_4_^−^	−39.9	−5.7	−34.3
**CS**·PF_6_^−^	−38.5	−6.0	−33.6
**Tc**·F^−^	−45.6	−6.0	−59.0
**Tc·**Cl^−^	−34.6	−3.8	−53.6
**Tc**·Br^−^	−32.5	−3.5	−51.0
**Tc**·I^−^	−32.6	−4.5	−47.1
**Tc·**CN^−^	−31.7	−3.3	−53.0
**Tc**·HF_2_^−^	−35.1	−1.8	−50.7
**Tc**·N_3_^−^	−33.7	−2.9	−46.7
**Tc**·SCN^−^	−28.3	−2.3	−46.7
**Tc**·NO_2_^−^	−33.4	−1.5	−48.3
**Tc**·NO_3_^−^	−36.1	−4.3	−45.2
**Tc**·BF_4_^−^	−31.5	−3.5	−46.1
**Tc**·ClO_4_^−^	−29.3	−3.2	−46.1
**Tc**·PF_6_^−^	−29.2	−2.9	−43.5

Across all three receptors, the binding free energies in DCM are notably lower than in the gas phase, emphasizing the impact of solvation in weakening electrostatic attraction [28, 44]. For smaller anions, binding strength (Δ*G*) generally decreases as cavity size increases, particularly for small, spherical anions like F^−^ and Cl^−^, though the extent of this trend depends on the dominant interaction types in each host–guest complex. For example, **CS**·F^−^ has only −6 kcal/mol relative to the **Tz**·F^−^ complex at −14 kcal/mol in DCM. Furthermore, fluoride exhibits strong binding with all three macrocycles due to its high charge density and strong electrostatic interactions.

For triazolophane, small anions are preferentially bound. In the gas phase, Cl^−^ and HF_2_^−^ exhibit comparably strong binding, while N_3_^−^ and I^−^ show moderate affinities. As expected, large anions that do not fit the cavity bind weakly. In DCM, the preference for small, spherical anions becomes more pronounced: F^−^ and Cl^−^ bind significantly more strongly than bulkier or more diffuse anions like I^−^ and PF_6_^−^. Although HF_2_^−^ binds comparably to Cl^−^ in the gas phase, it binds less strongly in solution, likely due to partial solvent exposure or reduced hydrogen bonding (Figure 4b). These trends confirm triazolophane as the most effective macrocycle for binding small, highly charged halides.

The larger cyanostar and tricarb receptors exhibit weaker binding overall compared to triazolophane, with tricarb showing consistently lower affinities than cyanostar across all anions. In the gas phase, both F^−^ and HF_2_^−^ exhibit the strongest binding for these two hosts. In DCM, however, F^−^ remains the strongest binder, while HF_2_^−^ binding is substantially reduced, especially for tricarb. Cyanostar shows relatively uniform binding across many anions in solution, with free energies between −4 and −6 kcal/mol for most guests. For the larger anions that are weakly bound by triazolophane, cyanostar and tricarb show a preference for I^−^ in solution, but show weak binding with 3D anions, such as BF_4_^−^, ClO_4_^−^, and PF_6_^−^. This underscores the anion recognition driven by size and shape matching [31].

In addition to binding free energies, the solvation energies of the complexes (Table 1) provide further insight into the role of the solvent environment. Across all three macrocycles, the solvation free energies are strongly negative, reflecting the stabilization of macrocycle–anion complexes in DCM relative to the gas phase. Fluoride and chloride complexes, in particular, exhibit the largest stabilization across all hosts, consistent with their high charge density and strong interactions with the polar solvent. In contrast, bulkier and more diffuse anions such as PF_6_^−^ or ClO_4_^−^ experience much weaker solvation effects. This trend indicates that solvation selectively dampens the binding of small, hard anions more than that of bulkier guests. Overall, solvation acts as a “leveling effect,” reducing the large differences in binding strength seen in the gas phase and underscoring the necessity of considering the solvent environment when evaluating receptor performance.

The SAPT analysis (Figure 7, Table S2) further elucidates the nature of these binding interactions. As shown by the energy breakdown, electrostatic interactions are the dominating attractive term in all complexes (Figure 7b,d,f). The contributions of exchange, induction, and dispersion vary depending on the anion and macrocycle. Triazolophane, with its compact and less flexible binding cavity, exhibits strong electrostatics. In contrast, cyanostar and tricarb display higher non‐electrostatic contributions due to their larger, more flexible cavities (Figure 7c,e). The exchange term increases with greater wavefunction overlap between the receptor and anion, which is particularly evident in triazolophane (Figure 7a). Its constrained cavity forces anions into close contact, leading to stronger repulsive forces.

### 3.2 2:1 Anion Binding Complexes

**FIGURE 8:**
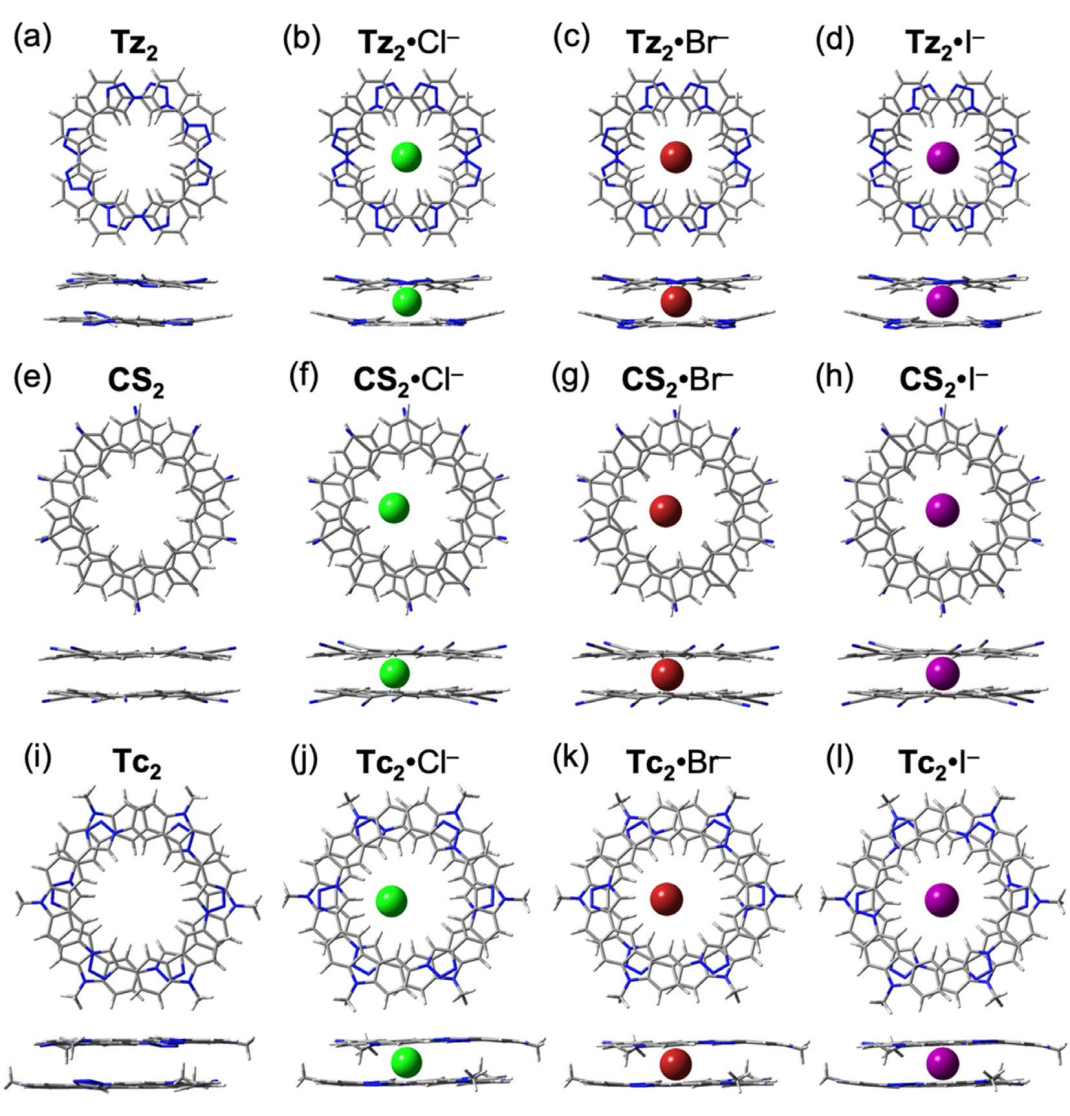
Top views (top row) and side (bottom row) views of (a) **Tz**_2_, (e) **CS**_2_, and (i) **Tc**_2_. Top views (top row) and side (bottom row) views of (b) **Tz**_2_·Cl^−^, (c) **Tz**_2_·Br^−^, (d) **Tz**_2_·I^−^, (f) **CS**_2_·Cl^−^, (g) **CS**_2_·Br^−^, (h) **CS**_2_·I^−^, (j) **Tc**_2_·Cl^−^, (k) **Tc**_2_·Br^−^, and (l) **Tc**_2_·I^−^.

While the macrocycles exhibit weak affinities with large anions in 1:1 binding modes, they can associate through π–π interactions to form 2:1 sandwich complexes [29, 30, 59]. This dimerization enhances anion encapsulation and contributes to the stabilization of the complex. The stacking of the macrocycle dimers is illustrated in Figure 8a,e,i, with interplanar distances of 3.35, 3.37, and 3.30 Å and rotation angles of 33°, 36°, and 55° for **Tz**_2_, **CS**_2_, and **Tc**_2_, respectively. In this study, we investigate the 2:1 binding of three halides: Cl^−^, Br^−^, and I^−^, as well as four traditionally “non‐coordinating” [60] polyatomic anions: SCN^−^, BF_4_^−^, ClO_4_^−^, and PF_6_^−^.

In the 2:1 complexes formed with halide anions, the anions reside within the dimer cavity, positioned between the mean planes of the two macrocycles. This geometry substantially enhances anion encapsulation [59], enabling effective binding of the larger iodide ion in **Tz**_2_·I^−^ (Figure 8d), in contrast to the 1:1 **Tz**·I^−^ complex (Figure 3d), where iodide could not fully penetrate the smaller cavity of the triazolophane macrocycle. Furthermore, smaller chloride and bromide ions exhibit off‐center positioning within the cyanostar (Figure 8f,g) and tricarb (Figure 8j,k) sandwich complexes, highlighting the macrocycles' preference for larger anions in the 2:1 binding mode.

**FIGURE 9:**
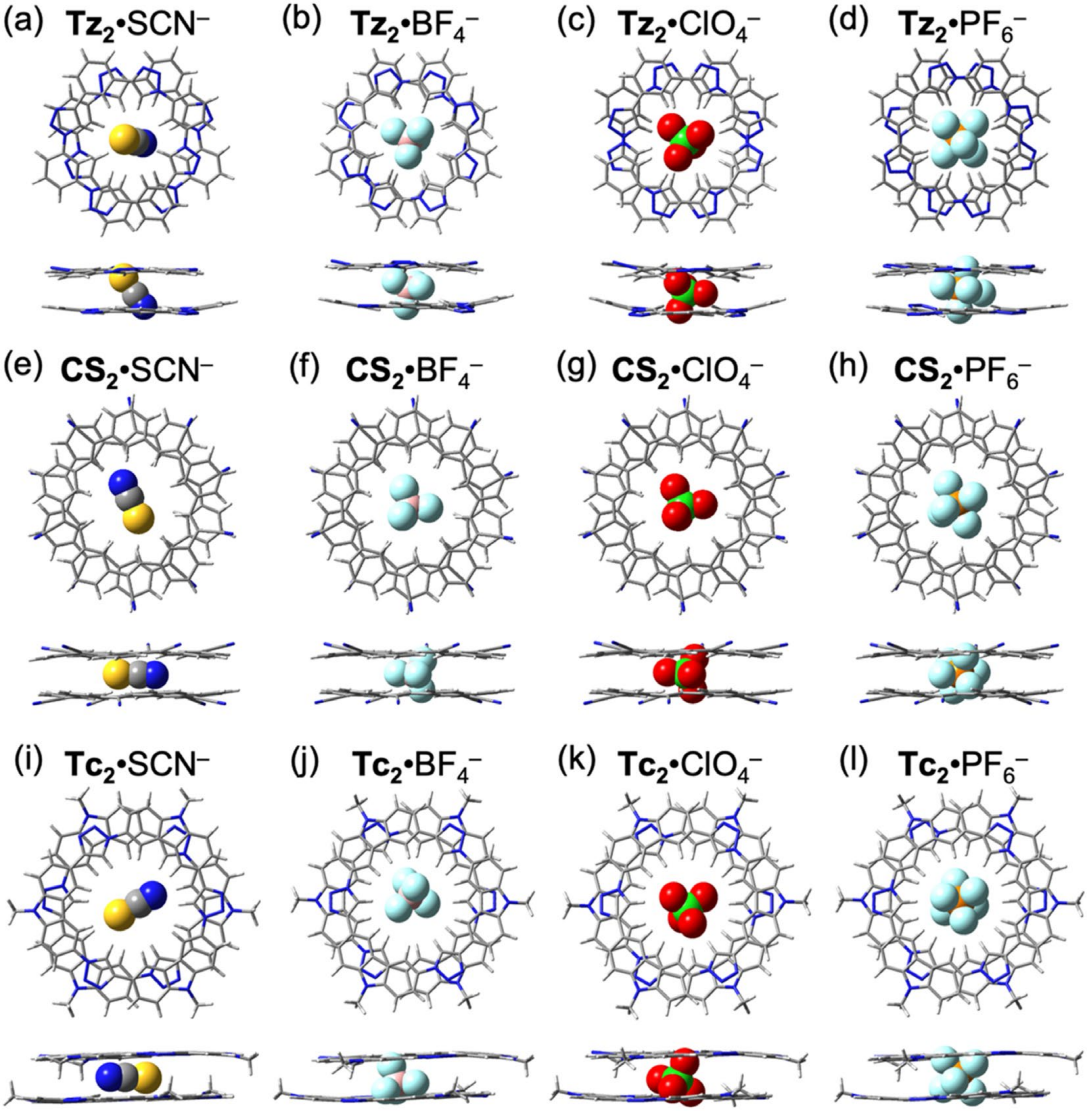
Optimized structures of 2:1 complexes formed with polyatomic anions: Top views (top row) and side (bottom row) views of (a) **Tz**_2_·SCN^−^, (b) **Tz**_2_·BF_4_^−^, (c) **Tz**_2_·ClO_4_^−^, (d) **Tz**_2_·PF_6_^−^, (e) **CS**_2_·SCN^−^, (f) **CS**_2_·BF_4_^−^, (g) **CS**_2_·ClO_4_^−^, (h) **Cs**_2_·PF_6_^−^, (i) **Tc**_2_·SCN^−^, (j) **Tc**_2_·BF_4_^−^, (k) **Tc**_2_·ClO_4_^−^, and (l) **Tc**_2_·PF_6_^−^.

Although the planar macrocycles failed to effectively encapsulate large polyatomic anions in 1:1 binding (*vide supra*), the formation of 2:1 sandwich complexes significantly enhances their stabilization (Figure 9). In these sandwich structures, the hydrogen bond acceptor atoms of the polyatomic anions are surrounded within the cavity by the macrocycles' CH donor groups, that is, triazoles in triazolophane and tricarb, and cyanoolefin groups in cyanostar. Notably, **Tz**_2_·PF_6_^−^ (Figure 9d) represents an exception, as the cavity formed by the triazolophane dimer remains insufficiently large to fully accommodate this particularly bulky anion.

The 2:1 anion binding of macrocycles can be characterized by two equilibria. The first involves the formation of a 1:1 complex between the receptor (**M**) and the anion (A^−^):

(1) $$M+A^{-}⇌M\cdot A^{-}\;∆G_{1:1},K_{1:1}$$

The second involves association of a second macrocycle to form the 2:1 sandwich complex (**M**_2_·A^−^):

(2) $$M\cdot A^{-}+M⇌M_{2}\cdot A^{-}\;∆G_{2:1},K_{2:1}$$

The overall binding process can be represented as:

(3) $$2M+A^{-}⇌M_{2}\cdot A^{-}\;∆G_{\text{overall}},\beta_{2:1}$$

Here, *K*_1:1_, *K*_2:1_, and *β*_2:1_ are the equilibrium constants for the respective reactions, with *β*_2:1_ representing the overall 2:1 binding affinity. To quantify the effect of the binding of the first macrocycle on the second, the cooperativity [61] coefficient α is defined as:

(4) $$\alpha =\frac{4K_{2:1}}{K_{1:1}}$$

A value of *α* > 1 indicates positive cooperativity, where the first macrocycle enhances binding of the second. Conversely, *α* < 1 implies negative cooperativity, where the first binding event hinders the second. When *α* = 1, the two binding events are non‐cooperative, meaning they occur independently [27, 62].

The calculated 2:1 binding free energies are summarized in Table 2, demonstrating consistently stronger binding compared to the 1:1 complexes. Triazolophane exhibits strong and selective binding for spherical halides, while cyanostar and tricarb show stronger binding with larger, polyatomic anions such as PF_6_^−^ and ClO_4_^−^. This trend is qualitatively consistent with experimental findings [29, 30, 41]. As expected, solvation in DCM significantly weakens binding for all receptors. However, the solvent‐induced decrease in binding free energy is generally less pronounced for 2:1 complexes, as the anion guest is more effectively shielded from the solvent environment within the sandwich structure [63].

**TABLE 2:** Calculated binding free energies (∆*G*, kcal/mol) for triazolophane, cyanostar, and tricarb in the gas phase and dichloromethane (DCM).

Macrocycle	Anion	${∆G}_{\text{overall}}^{\mathrm{gas}}$	${∆G}_{1:1}^{\mathrm{gas}}$	${∆G}_{2:1}^{\mathrm{gas}}$	${∆G}_{\text{overall}}^{\mathrm{DCM}}$	${∆G}_{1:1}^{\mathrm{DCM}}$	${∆G}_{2:1}^{\mathrm{DCM}}$
**Tz**	Cl^−^	−77.5	−54.2	−23.3	−18.9	−11.8	−7.1
	Br^−^	−74.7	−50.7	−24.0	−18.3	−10.1	−8.2
	I^−^	−70.6	−43.1	−27.5	−18.3	−7.4	−10.9
	SCN^−^	−67.0	−39.5	−27.5	−16.2	−7.4	−8.8
	BF_4_^−^	−62.7	−39.3	−23.4	−12.6	−5.8	−6.8
	ClO_4_^−^	−59.9	−37.2	−22.7	−10.4	−5.6	−4.8
	PF_6_^−^	−54.2	−36.3	−17.9	−6.1	−6.3	0.2
**CS**	Cl^−^	−69.2	−45.2	−24.0	−21.5	−5.9	−15.6
	Br^−^	−66.8	−44.3	−22.5	−21.7	−5.9	−15.8
	I^−^	−68.7	−42.2	−26.5	−23.1	−6.8	−16.3
	SCN^−^	−63.2	−37.1	−26.1	−20.9	−4.7	−16.2
	BF_4_^−^	−64.0	−40.7	−23.3	−20.8	−5.6	−15.2
	ClO_4_^−^	−64.3	−39.9	−24.4	−22.3	−5.7	−16.6
	PF_6_^−^	−63.4	−38.5	−24.9	−23.4	−6.0	−17.4
**Tc**	Cl^−^	−45.9	−34.6	−11.3	−10.2	−3.8	−6.4
	Br^−^	−43.4	−32.5	−10.9	−9.7	−3.5	−6.2
	I^−^	−43.7	−32.6	−11.1	−11.8	−4.5	−7.3
	SCN^−^	−41.0	−28.3	−12.7	−10.2	−2.3	−7.9
	BF_4_^−^	−41.9	−31.5	−10.4	−10.7	−3.5	−7.2
	ClO_4_^−^	−41.1	−29.3	−11.8	−10.6	−3.2	−7.4
	PF_6_^−^	−39.8	−29.2	−10.6	−11.2	−2.9	−8.3

**FIGURE 10:**
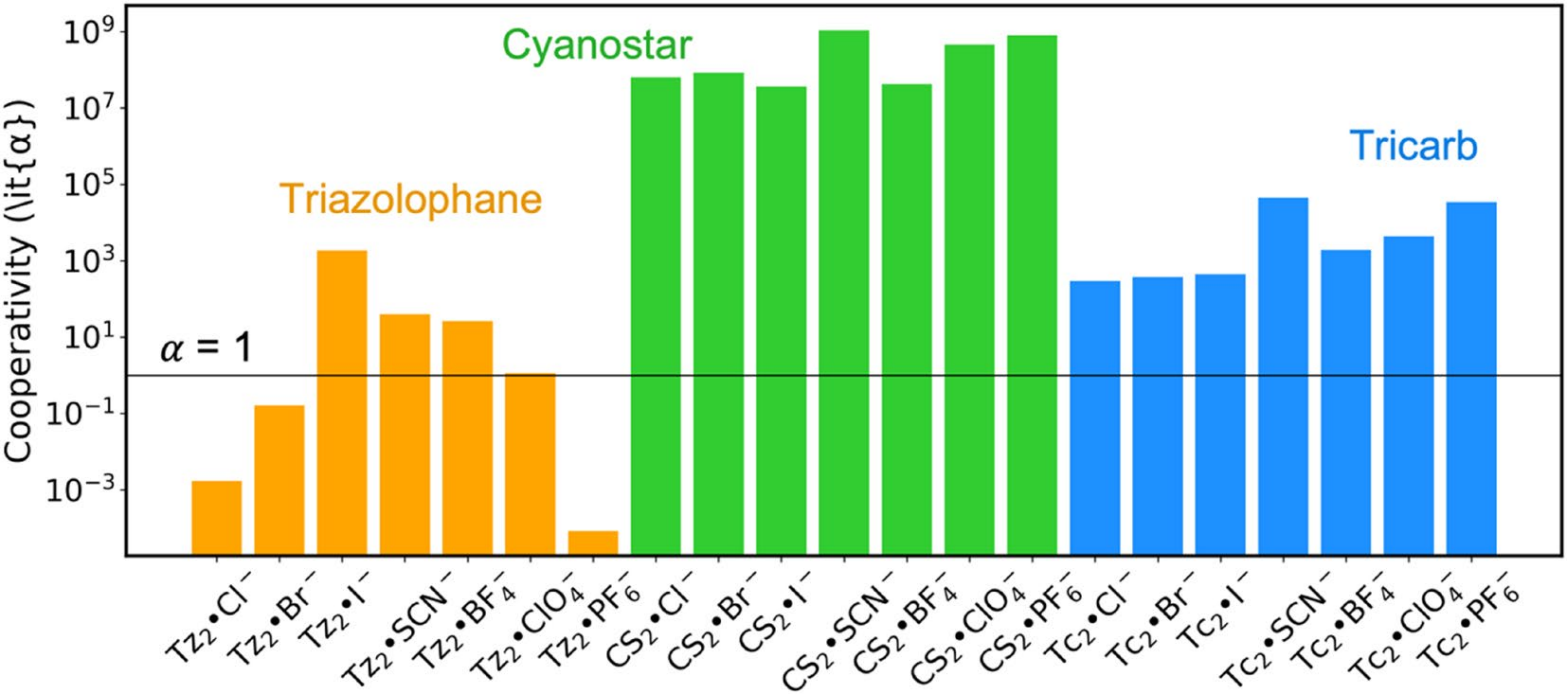
Cooperativity of 2:1 binding for triazolophane, cyanostar, and tricarb. *α* = 1 indicates non‐cooperative binding.

The cooperativity (*α*) of 2:1 binding for triazolophane, cyanostar, and tricarb in DCM is presented in Figure 10 and Table S4. For triazolophane, PF_6_^−^ exhibits a notably low cooperativity factor, reflecting steric crowding within this receptor's compact binding pocket. Negative cooperativity is also observed for Cl^−^ and Br^−^, where the small size of both the receptor and the anions favors tight 1:1 binding that may hinder a second binding event. Particularly, the rank order of Cl^−^, Br^−^, and I^−^ matches the experimental trend reported by Li et al. [59] for pyridyl‐containing triazolophanes. In contrast, ClO_4_^−^ shows non‐cooperativity while SCN^−^ and BF_4_^−^ all show positive cooperativity.

For the larger cyanostar and tricarb macrocycles, all anions examined exhibit positive cooperativity, highlighting the propensity of the two larger macrocycles to form sandwich complexes stabilized by π–π stacking. Interestingly, the computed cooperativity factors for cyanostar are approximately twice as large as those for tricarb. This differs from the experiment, where tricarb displays much stronger positive cooperativity than cyanostar [30]. One explanation is tricarb's pronounced self‐association, which is about one million times greater than that of cyanostar. This effect is enhanced by solvophobic forces and is entropically favored [64], but cannot be captured by a continuum model, likely contributing to the discrepancy with our computational predictions.

Additionally, because of the substantial size of the 2:1 sandwich complexes, we employed a lower level of theory for the geometry optimizations compared to the 1:1 calculations, which could lead to the loss of accuracy. It is also important to note that the experimental binding free energies were measured in different solvents to enhance macrocycle solubility, for example, in 40% MeOH/DCM for cyanostar [29] and 20% MeOH/CHCl_3_ for tricarb [30]. When extrapolated to the dielectric constant of dichloromethane (*ε*_r_ = 8.93), our calculation systematically underestimates the binding free energies.

**FIGURE 11:**
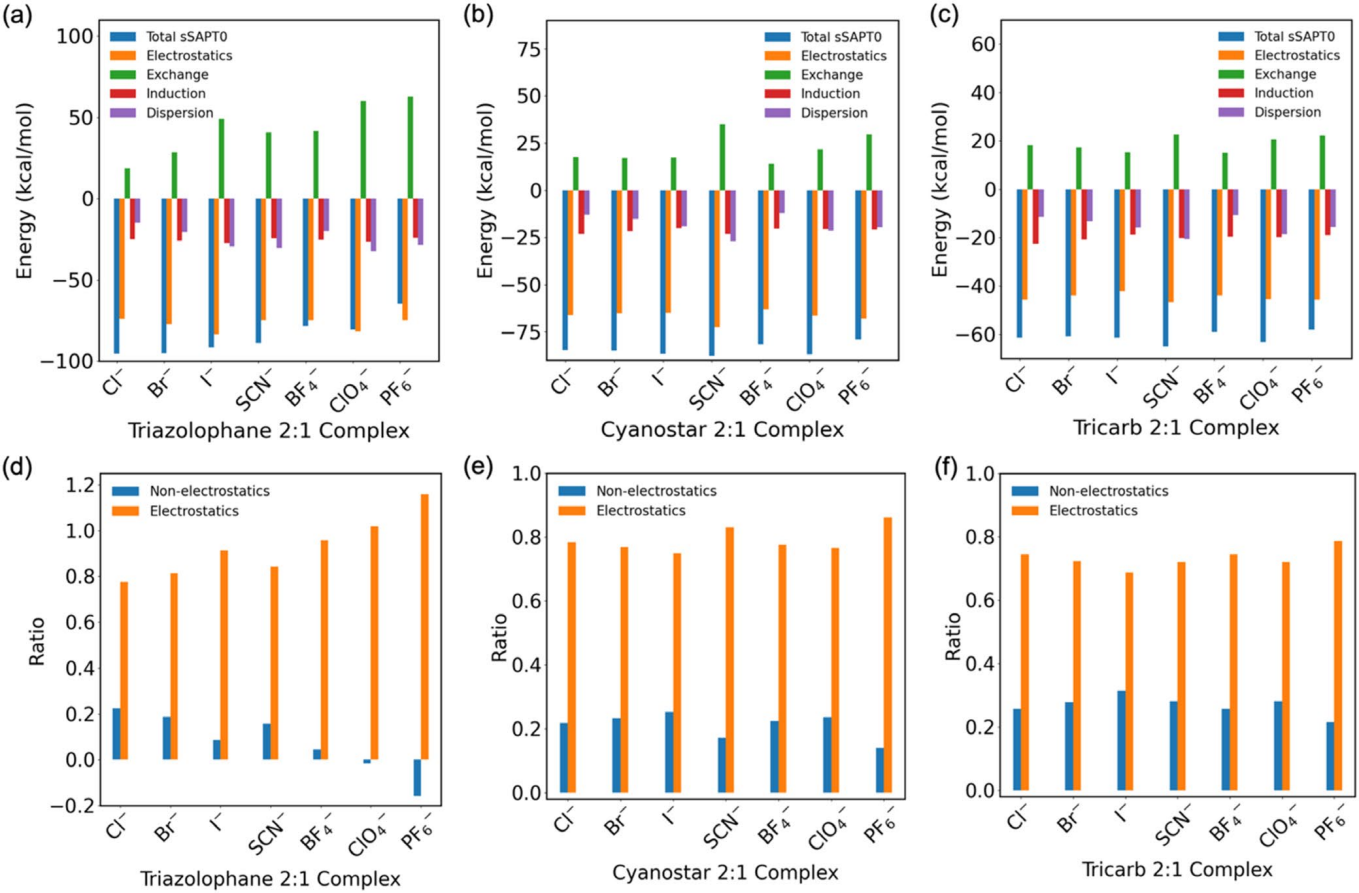
SAPT energy decomposition of 2:1 complexes for (a) triazolophane, (b) cyanostar, and (c) tricarb. Ratios of non‐electrostatics (exchange‐repulsion, induction and dispersion) and electrostatics of the total sSAPT0 energies for (d) triazolophane, (e) cyanostar, and (f) tricarb.

SAPT energy decomposition further supports the trends in binding energies (Table S3), highlighting the roles of electrostatics, exchange‐repulsion, induction, and dispersion interactions (Figure 11). Electrostatics remain the dominant attractive interaction (Figure 11d–f), although higher contributions from non‐electrostatics are found relative to 1:1 complexes. High exchange repulsion is noted in **Tz**_2_·ClO_4_^−^ and **Tz**_2_·PF_6_^−^ (Figure 11a), suggesting wavefunction overlap between the triazolophane dimers and the large anions. BF_4_^−^ complexes show the weakest dispersion interaction (Figure 11a–c), which aligns with its smallest size and lowest polarizability among the four. Overall, the 2:1 binding mode proves to be an effective strategy to coordinate the traditionally weak binding large anions.

## 4 Conclusions

The anion binding properties of three macrocyclic receptors, triazolophane, cyanostar and tricarb, bearing CH hydrogen bond donors were investigated computationally. Using a diverse set of 13 anions spanning a wide range of sizes and shapes, we evaluated the selectivity of these receptors in the 1:1 binding mode. Due to their preorganized, shape‐persistent cavities, the macrocycles favor anions that fit within their planar binding sites in solution. Specifically, triazolophane exhibits a preference for fluoride and chloride, while cyanostar favor iodide. For weakly coordinating polyatomic anions that exceed the 2D shape of the cavity, the macrocycles tend to dimerize, forming 2:1 sandwich complexes. Energy decomposition analysis confirmed that electrostatics is the primary stabilizing force in both binding modes. These findings highlight the critical role of cavity size and electrostatic interactions in governing anion selectivity and binding strength. The ability of macrocycles to switch between 1:1 and 2:1 binding modes offers a versatile strategy for recognizing diverse anions, particularly those traditionally considered weakly coordinating. This insight provides a foundation for the rational design of next‐generation anion receptors with enhanced selectivity and tunable binding properties.

## Conflicts of Interest

The authors declare no conflicts of interest.

## Supporting information


**Table S1:** Geometry and size [1] of the anions.
**Table S2:** Energy decomposition of 1:1 complexes formed with macrocycle receptors in the gas phase (kcal/mol).
**Table S3:** Energy decomposition of 2:1 complexes formed with macrocycle receptors in the gas phase (kcal/mol).
**Table S4:** Cooperativity of 2:1 complexes in DCM.

## Data Availability

The xyz atomic coordinates of the macrocycles and anion‐macrocycle complexes investigated in this study are included in the file “atomic_coordinates.zip”.
