# Supplementary material for: Anion Binding by Macrocyclic Receptors: Computational Landscape of 1:1 and 2:1 Stoichiometries
Source: J Comput Chem. 2025 Nov 14;46(30):e70270. doi: 10.1002/jcc.70270 (PMC12617332; doi:10.1002/jcc.70270)
Supplement: Supplementary file 1 — Table S1: Geometry and size [1] of the anions. Table S2: Energy decomposition of 1:1 complexes formed with macrocycle receptors in the gas phase (kcal/mol). Table S3: Energy decomposition of 2:1 complexes formed with macrocycle receptors in the gas phase (kcal/mol). Table S4: Cooperativity of 2:1 complexes in DCM. [file JCC-46-0-s001.docx]

***Supporting Information***

**Anion Binding by Macrocyclic Receptors: Computational Landscape of 1:1 and 2:1 Stoichiometries**

*Minwei Che, Amar H. Flood, and Krishnan Raghavachari**

Department of Chemistry, Indiana University, Bloomington IN 47405, USA

**Table of Contents**

|  |  |
| --- | --- |
| Size of Selected Anions…………………………………………………………………… | S3 |
| SAPT Energy Decomposition …………………………………………………………… | S4 |
| Cooperativity……………………………………………………………………………… | S6 |
| Coefficients of the custom basis set used for Iodine. ………………………………….… | S7 |
| References………………………………………………………………………………… | S9 |
|  |  |
|  |  |
|  |  |
|  |  |
|  |  |
|  |  |
|  |  |
|  |  |

**Size of Selected Anions**

Table S1. Geometry and size^1^ of the anions.

| Anion | Geometry | Diameter (Å) |
| --- | --- | --- |
| F^−^ | spherical | 2.5 |
| Cl^−^ | spherical | 3.4 |
| Br^−^ | spherical | 3.8 |
| I^−^ | spherical | 4.2 |
| CN^−^ | linear | 3.7 |
| HF_2_^−^ | linear | 3.4 |
| N_3_^−^ | linear | 3.9 |
| SCN^−^ | linear | 4.2 |
| NO_2_^−^ | planar | 3.7 |
| NO_3_^−^ | planar | 4.0 |
| BF_4_^−^ | tetrahedral | 4.0 |
| ClO_4_^−^ | tetrahedral | 4.5 |
| PF_6_^−^ | octahedral | 4.8 |

**SAPT Energy Decomposition**

Table S2. Energy decomposition of 1:1 complexes formed with macrocycle receptors in the gas phase (kcal/mol).

| Complex | Total SAPT0 | Electrostatics | Exchange | Induction | Dispersion |
| --- | --- | --- | --- | --- | --- |
| **Tz**•F^−^ | −77.51 | −71.10 | 33.36 | −33.41 | −6.35 |
| **Tz**•Cl^−^ | −67.84 | −59.44 | 26.21 | −23.36 | −11.25 |
| **Tz**•Br^−^ | −63.88 | −62.19 | 37.88 | −24.31 | −15.26 |
| **Tz**•I^−^ | −55.16 | −58.10 | 43.58 | −23.10 | −17.52 |
| **Tz**•CN^−^ | −69.73 | −69.19 | 42.84 | −27.86 | −15.52 |
| **Tz**•HF_2_^−^ | −69.55 | −76.67 | 47.98 | −30.57 | −10.28 |
| **Tz**•N_3_^−^ | −65.40 | −69.23 | 50.72 | −27.48 | −19.40 |
| **Tz**•SCN^−^ | −54.43 | −48.84 | 31.15 | −20.19 | −16.56 |
| **Tz**•NO_2_^−^ | −68.24 | −71.05 | 47.14 | −28.09 | −16.23 |
| **Tz**•NO_3_^−^ | −59.85 | −59.81 | 39.33 | −23.61 | −15.76 |
| **Tz**•BF_4_^−^ | −50.24 | −50.90 | 32.72 | −20.05 | −12.01 |
| **Tz**•ClO_4_^−^ | −50.30 | −49.40 | 36.29 | −19.84 | −17.36 |
| **Tz**•PF_6_^−^ | −43.48 | −41.27 | 26.55 | −16.02 | −12.74 |
| **CS**•F^−^ | −67.11 | −60.56 | 30.60 | −31.46 | −5.70 |
| **CS**•Cl^−^ | −56.88 | −44.95 | 13.73 | −18.10 | −7.56 |
| **CS**•Br^−^ | −56.76 | −43.37 | 12.03 | −16.53 | −8.89 |
| **CS**•I^−^ | −55.42 | −46.07 | 20.95 | −17.17 | −13.14 |
| **CS**•CN^−^ | −60.16 | −47.45 | 15.77 | −19.16 | −9.33 |
| **CS**•HF_2_^−^ | −61.46 | −53.15 | 20.49 | −22.45 | −6.35 |
| **CS**•N_3_^−^ | −63.77 | −52.24 | 24.02 | −21.03 | −14.53 |
| **CS**•SCN^−^ | −54.59 | −48.64 | 28.32 | −18.32 | −15.95 |
| **CS**•NO_2_^−^ | −64.57 | −50.91 | 16.72 | −20.27 | −10.11 |
| **CS**•NO_3_^−^ | −63.28 | −54.59 | 26.74 | −21.70 | −13.73 |
| **CS**•BF_4_^−^ | −54.13 | −45.09 | 16.82 | −17.12 | −8.74 |
| **CS**•ClO_4_^−^ | −55.25 | −47.69 | 25.08 | −17.77 | −14.88 |
| **CS**•PF_6_^−^ | −47.55 | −42.21 | 23.56 | −16.05 | −12.84 |
| **Tc**•F^−^ | −54.20 | −50.88 | 34.48 | −32.36 | −5.43 |
| **Tc•**Cl^−^ | −43.72 | −33.26 | 13.55 | −17.29 | −6.72 |
| **Tc**•Br^−^ | −43.02 | −31.75 | 12.31 | −15.81 | −7.77 |
| **Tc**•I^−^ | −42.79 | −31.33 | 13.53 | −15.04 | −9.94 |
| **Tc•**CN^−^ | −47.22 | −36.90 | 17.01 | −18.86 | −8.47 |
| **Tc**•HF_2_^−^ | −49.39 | −43.71 | 22.78 | −22.49 | −5.97 |
| **Tc**•N_3_^−^ | −50.94 | −40.30 | 21.75 | −20.18 | −12.21 |
| **Tc**•SCN^−^ | −44.58 | −39.18 | 27.40 | −18.60 | −14.21 |
| **Tc**•NO_2_^−^ | −50.89 | −39.38 | 17.06 | −19.91 | −8.66 |

Table S2 (continued). Energy decomposition of 1:1 complexes formed with macrocycle receptors in the gas phase (kcal/mol).

| Complex | Total SAPT0 | Electrostatics | Exchange | Induction | Dispersion |
| --- | --- | --- | --- | --- | --- |
| **Tc**•NO_3_^−^ | −52.13 | −40.19 | 17.67 | −19.06 | −10.54 |
| **Tc**•BF_4_^−^ | −43.32 | −36.31 | 17.32 | −16.74 | −7.59 |
| **Tc**•ClO_4_^−^ | −43.93 | −33.32 | 17.04 | −15.85 | −11.80 |
| **Tc**•PF_6_^−^ | −38.45 | −30.07 | 16.16 | −14.60 | −9.94 |

Table S3. Energy decomposition of 2:1 complexes formed with macrocycle receptors in the gas phase (kcal/mol).

| Complex | Total SAPT0 | Electrostatics | Exchange | Induction | Dispersion |
| --- | --- | --- | --- | --- | --- |
| **Tz**_2_•Cl^−^ | −95.40 | −74.01 | 18.56 | −25.09 | −14.86 |
| **Tz**_2_•Br^−^ | −95.06 | −77.35 | 28.53 | −25.86 | −20.39 |
| **Tz**_2_•I^−^ | −91.53 | −83.61 | 48.98 | −27.47 | −29.44 |
| **Tz**_2_•SCN^−^ | −88.89 | −74.98 | 40.79 | −24.30 | −30.39 |
| **Tz**_2_•BF_4_^−^ | −78.33 | −74.93 | 41.63 | −25.16 | −19.88 |
| **Tz**_2_•ClO_4_^−^ | −80.47 | −81.86 | 60.15 | −26.36 | −32.40 |
| **Tz**_2_•PF_6_^−^ | −64.77 | −75.02 | 62.90 | −24.05 | −28.60 |
| **CS**_2_•Cl^−^ | −84.66 | −66.21 | 17.62 | −23.20 | −12.87 |
| **CS**_2_•Br^−^ | −84.96 | −65.17 | 16.93 | −21.55 | −15.17 |
| **CS**_2_•I^−^ | −86.64 | −64.79 | 17.41 | −20.20 | −19.06 |
| **CS**_2_•SCN^−^ | −87.62 | −72.63 | 35.04 | −23.14 | −26.90 |
| **CS**_2_•BF_4_^−^ | −81.49 | −63.23 | 14.02 | −20.22 | −12.05 |
| **CS**_2_•ClO_4_^−^ | −86.82 | −66.43 | 21.60 | −20.72 | −21.26 |
| **CS**_2_•PF_6_^−^ | −78.87 | −67.89 | 29.47 | −20.82 | −19.63 |
| **Tc**_2_•Cl^−^ | −61.41 | −45.66 | 18.28 | −22.66 | −11.37 |
| **Tc**_2_•Br^−^ | −60.81 | −43.96 | 17.15 | −20.77 | −13.22 |
| **Tc**_2_•I^−^ | −61.45 | −42.17 | 15.31 | −18.75 | −15.83 |
| **Tc**_2_•SCN^−^ | −64.97 | −46.76 | 22.53 | −20.20 | −20.53 |
| **Tc**_2_•BF_4_^−^ | −59.06 | −43.90 | 15.05 | −19.52 | −10.69 |
| **Tc**_2_•ClO_4_^−^ | −63.21 | −45.52 | 20.65 | −19.78 | −18.56 |
| **Tc**_2_•PF_6_^−^ | −58.13 | −45.65 | 22.25 | −19.07 | −15.66 |

**Cooperativity**

Table S4. Cooperativity of 2:1 complexes in DCM.

|  | Cooperativity (*α*) |
| --- | --- |
| **Tz**_2_•Cl^−^ | 1.72 × 10^−3^ |
| **Tz**_2_•Br^−^ | 1.62 × 10^−1^ |
| **Tz**_2_•I^−^ | 1.83 × 10^3^ |
| **Tz**_2_•SCN^−^ | 4.01 × 10^1^ |
| **Tz**_2_•BF_4_^−^ | 2.60 × 10^1^ |
| **Tz**_2_•ClO_4_^−^ | 1.14 × 10^0^ |
| **Tz**_2_•PF_6_^−^ | 8.39 × 10^−5^ |
| **CS**_2_•Cl^−^ | 6.22 × 10^7^ |
| **CS**_2_•Br^−^ | 8.33 × 10^7^ |
| **CS**_2_•I^−^ | 3.70 × 10^7^ |
| **CS**_2_•SCN^−^ | 1.06 × 10^9^ |
| **CS**_2_•BF_4_^−^ | 4.11 × 10^7^ |
| **CS**_2_•ClO_4_^−^ | 4.47 × 10^8^ |
| **CS**_2_•PF_6_^−^ | 7.88 × 10^8^ |
| **Tc**_2_•Cl^−^ | 2.93 × 10^2^ |
| **Tc**_2_•Br^−^ | 3.75 × 10^2^ |
| **Tc**_2_•I^−^ | 4.44 × 10^2^ |
| **Tc**_2_•SCN^−^ | 4.43 × 10^4^ |
| **Tc**_2_•BF_4_^−^ | 1.88 × 10^3^ |
| **Tc**_2_•ClO_4_^−^ | 4.33 × 10^3^ |
| **Tc**_2_•PF_6_^−^ | 3.37 × 10^4^ |

**Iodine 6-311+G(d) Basis Set Information**

The following coefficients are formatted to be used in the *Gaussian16* program.^2^

I 0

S 5 1.00

444750.0 0.00089

66127.00 0.00694

14815.00 0.03609

4144.900 0.13568

1361.200 0.33878

S 2 1.00

508.4400 0.43659

209.5900 0.18375

S 1 1.00

81.959 1.00000

S 1 1.00

36.805 1.00000

S 1 1.00

13.495 1.00000

S 1 1.00

6.8859 1.00000

S 1 1.00

2.5520 1.00000

S 1 1.00

1.2088 1.00000

S 1 1.00

0.2734 1.00000

S 1 1.00

0.1009 1.00000

S 1 1.00

0.025225 1.00000

P 4 1.00

2953.600 0.01221

712.6100 0.08587

236.7100 0.29493

92.63100 0.47849

P 1 1.00

39.73200 1.00000

P 1 1.00

17.27300 1.000000

P 1 1.00

7.957000 1.000000

P 1 1.00

3.152900 1.000000

P 1 1.00

1.332800 1.000000

P 1 1.00

0.494700 1.000000

P 1 1.00

0.216000 1.000000

P 1 1.00

0.082930 1.000000

P 1 1.00

0.0207325 1.000000

D 3 1.00

261.9500 0.03144

76.73400 0.19028

27.55100 0.47247

D 1 1.00

10.60600 1.000000

D 1 1.00

3.421700 1.000000

D 1 1.00

1.137000 1.000000

D 1 1.00

0.302000 1.000000

****

**References**

1. Roobottom, H. K.; Jenkins, H. D. B.; Passmore, J.; Glasser, L. Journal of Chemical Education 1999, 76(11), 1570-1573.

2. Frisch, M. J.; Trucks, G. W.; Schlegel, H. B.; Scuseria, G. E.; Robb, M. A.; Cheeseman, J. R.; Scalmani, G.; Barone, V.; Petersson, G. A.; Nakatsuji, H.; Li, X.; Caricato, M.; Marenich, A. V.; Bloino, J.; Janesko, B. G.; Gomperts, R.; Mennucci, B.; Hratchian, H. P.; Ortiz, J. V.; Izmaylov, A. F.; Sonnenberg, J. L.; Williams-Young, D.; Ding, F.; Lipparini, F.; Egidi, F.; Goings, J.; Peng, B.; Petrone, A.; Henderson, T.; Ranasinghe, D.; Zakrzewski, V. G.; Gao, J.; Rega, N.; Zheng, G.; Liang, W.; Hada, M.; Ehara, M.; Toyota, K.; Fukuda, R.; Hasegawa, J.; Ishida, M.; Nakajima, T.; Honda, Y.; Kitao, O.; Nakai, H.; Vreven, T.; Throssell, K.; Montgomery Jr., J. A.; Peralta, J. E.; Ogliaro, F.; Bearpark, M. J.; Heyd, J. J.; Brothers, E. N.; Kudin, K. N.; Staroverov, V. N.; Keith, T. A.; Kobayashi, R.; Normand, J.; Raghavachari, K.; Rendell, A. P.; Burant, J. C.; Iyengar, S. S.; Tomasi, J.; Cossi, M.; Millam, J. M.; Klene, M.; Adamo, C.; Cammi, R.; Ochterski, J. W.; Martin, R. L.; Morokuma, K.; Farkas, O.; Foresman, J. B.; Fox, D. J.: Wallingford, CT, 2016.
